# Large Temperature‐invariant Anomalous Nernst Effect in Non‐collinear Antiferromagnet Mn_3_Pt

**DOI:** 10.1002/advs.75880

**Published:** 2026-05-29

**Authors:** Pengwei Gong, Xiaolin Zhang, Wei Zhu, Mingzhi Wang, Chang Pan, Aoqi Xu, Yandong Guo, Yuheng Li, Jiaxin Chen, Yanchang Zhou, Zhong Shi, Dingfu Shao, Liang Liu, Shiming Zhou, Yicheng Guan, Ruiqing Cheng, Xuepeng Qiu

**Affiliations:** ^1^ School of Physics Science and Engineering Tongji University Shanghai China; ^2^ School of Physics and Technology Wuhan University Wuhan China; ^3^ Tsung‐Dao Lee Institute Shanghai Jiao Tong University Shanghai China; ^4^ Key Laboratory of Materials Physics Institute of Solid State Physics Hefei Institutes of Physical Science Chinese Academy of Sciences Hefei China

**Keywords:** antiferromagnetism, berry connection and curvatur, condensed matter physics, magnetic field, magnetization, materials science, nernst effect, physics, spintronics

## Abstract

The pursuit of next‐generation spintronic devices is pivoting toward noncollinear antiferromagnets (nc‐AFMs), their vanishing net magnetization and inherent ultrafast spin dynamics enable the development of faster and more energy‐efficient spintronic devices with strong resilience against external magnetic fields. Most importantly, their unique electronic and spin structures give rise to significant Berry curvature, leading to a significant magnetoelectric signal, such as the anomalous Nernst effect (ANE), even in the absence of sizable magnetization. Here, we report the discovery of a giant and remarkably temperature‐invariant ANE in high‐quality epitaxial Mn_3_Pt thin films with various compositions, which allows thermoelectric devices with a wide operating temperature window. Mn_3_Pt overwhelms other nc‐AFMs rivals with a significant ANE coefficient up to 0.71 µV/K at room temperature. Our combined experimental observation and theoretical calculations establish that the composition‐tunable Mn‐3d orbital states govern the Berry curvature landscape, responsible for this exceptional performance. This study highlights the composition tunability as a venue for applicable nc‐AFM spintronic devices.

## Graphical abstract

Based on the noncollinear antiferromagnetic Mn_3_Pt thin films, the composition‐tunable Mn‐3d orbital states directly govern the Berry curvature. As a result, a giant and temperature‐invariant anomalous Nernst coefficient of 0.71 µV/K is achieved under Mn‐rich conditions. This remarkable performance across a wide temperature range highlights its great potential for robust thermoelectric spintronic applications.

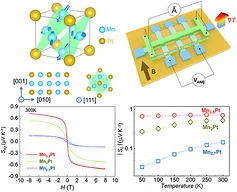

## 1 Introduction

The anomalous Nernst effect (ANE), a thermoelectric phenomenon primarily driven by the Berry curvature of electronic bands, has emerged as a vital research frontier bridging topology, magnetism, and thermoelectric applications [1, 2, 3]. Its unique advantages, including the ability to function without external magnetic fields, sensitivity to topological properties, and applicability in materials with no net magnetization, establish the ANE as a highly promising cornerstone for building next‐generation, low‐power, high‐performance technologies in both information processing and energy harvesting [4, 5, 6, 7, 8]. The study of the ANE has so far primarily centered on ferromagnetic materials, as their strong spontaneous magnetization facilitates a large signal [9, 10]. Despite sizable anomalous Nernst coefficients (*S_ij_*) (∼6 µV/K) reported in various ferromagnetic systems such as Heusler alloy [11, 12], iron‐based binaries [13, 14, 15], and Kagome magnets [16], ferromagnets suffer from inherent limitations. Their pronounced stray fields can interfere with adjacent components, and their small magnetic anisotropy renders devices sensitive to external disturbances [17, 18]. Most critically, the detectable ANE signal in ferromagnets is confined to a narrow temperature window below the Curie temperature, severely restricting the application perspective of the devices based on these materials [19, 20]. These fundamental constraints urge the need to explore alternative material platforms for robust thermoelectric devices [21].

Antiferromagnets have emerged as a promising candidate to overcome these limitations. Their key advantage lies in the absence of net magnetization, which fundamentally eliminates stray fields and grants them immunity to external magnetic interference [22, 23, 24, 25, 26, 27, 28, 29, 30]. Moreover, their magnetic dynamics reside in the terahertz frequency range, enabling ultrafast operations in the time scale of picosecond [31, 32, 33]. These combined properties make antiferromagnets superior to conventional ferromagnets. However, the very absence of net magnetization posed a longstanding challenge for a sizable signal from ANE [34]. This paradigm is overturned by the discovery of noncollinear antiferromagnets (nc‐AFM) [35, 36, 37, 38, 39, 40, 41, 42]. Crucially, their unique spin texture (Mn_3_X, X = Sn, Ge) breaks time‐reversal symmetry, generating a nontrivial Berry curvature distribution in *k*‐space [43, 44, 45, 46, 47, 48]. Here, the ANE is no longer generated from a net magnetic order but from the material's intrinsic topological order, enabling a detectable signal. The observation of a large ANE in nc‐AFM is first reported in Mn_3_Sn single crystals grown by the melt method, exhibiting a magnitude *S_ij_* of about 0.35 µV/K at 300 K [49]. Further progress is achieved with Mn_3_Ge, where *S_ij_* reached approximately 0.42 µV/K at 300 K, reinforcing the potential of nc‐AFM for ANE application [50]. These results also establish that a primary route for enhancing the ANE lies in identifying antiferromagnetic materials with regions of high Berry curvature near the Fermi surface [51, 52, 53].

To date, major advances in the ANE have been achieved in hexagonal close‐packed (HCP) systems such as Mn_3_Sn and Mn_3_Ge, where lower crystal symmetry facilitates the detection of pronounced topological responses like the ANE and the anomalous Hall effect (AHE). By contrast, the cubic systems, which served as the earliest theoretical platform, have proven more challenging for experimental observation, as their higher symmetry tends to suppress measurable signals of ANE [54]. Moreover, research across all these materials has predominantly focused on bulk crystals, while the ANE in nc‐AFM thin films remains largely unexplored, a critical gap that impedes the development of thermal microelectronics [55, 56, 57, 58, 59, 60]. To bridge this structural and functional gap, we introduce a Mn‐doping strategy in epitaxial thin films of the cubic nc‐AFM Mn_3_Pt, in order to enhance its Berry curvature. This approach yields a giant ANE coefficient. *S_ij_* up to 0.71 µV/K. Most interestingly, ANE coefficient which shows very small variation in a wide temperature range from 50 to 300 K can be achieved.

## 2 Results and Discussion

**FIGURE 1:**
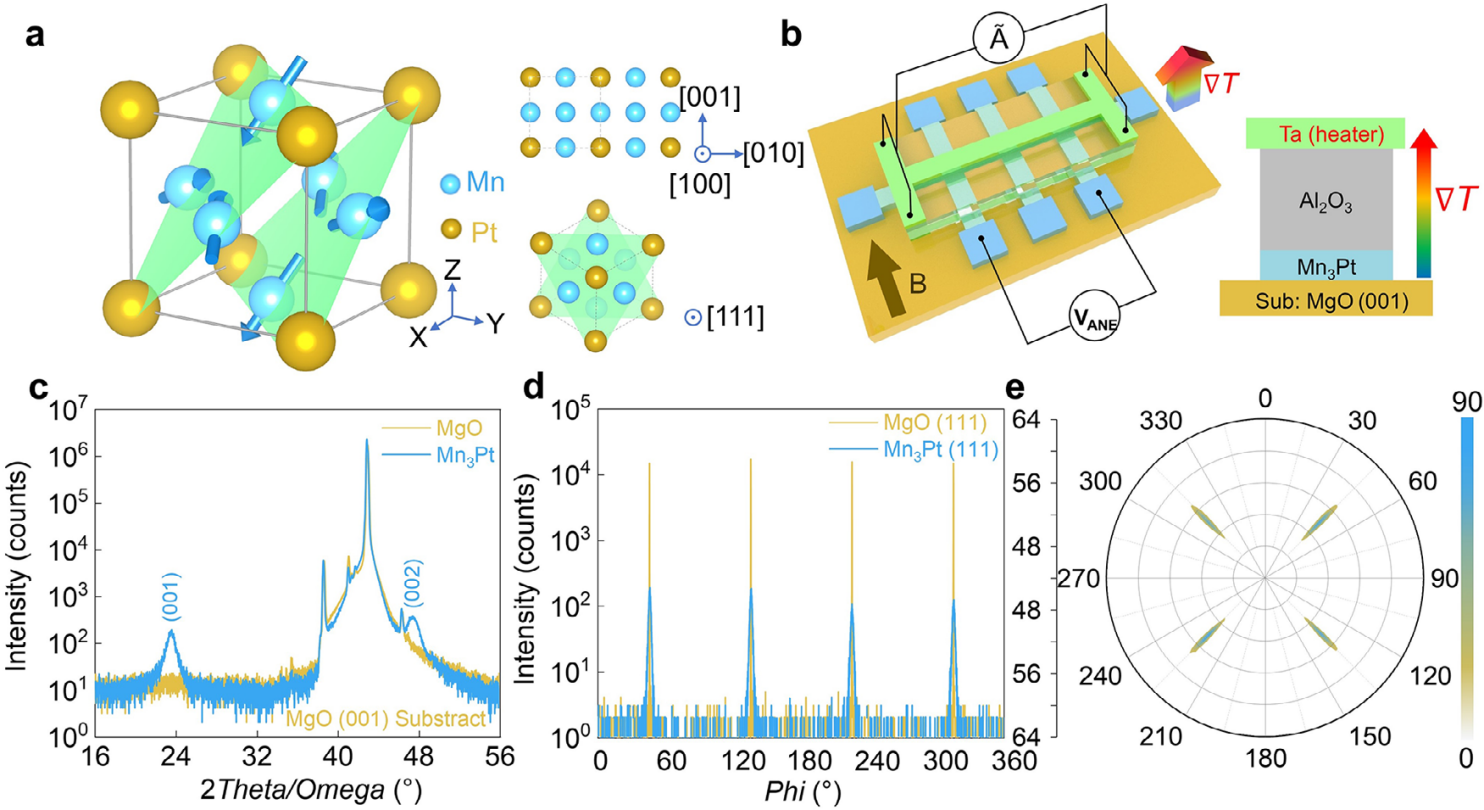
(a) Schematic illustration of the crystal structure of Mn_3_Pt. (b) Schematic of the measurement device geometry. (c) Out‐of‐plane *θ*‐2*θ* X‐ray diffraction patterns for the Mn_3_Pt (001) film and MgO (001) substrate. (d) *φ* scans of the Mn_3_Pt (111) and MgO (111) reflections. (e) Polar plot of the Mn_3_Pt (111) *φ*‐scan data.

Mn_3_Pt is a prototypical nc‐AFM that crystallizes in the face‐centered cubic (*fcc*) structure with an *L*1_2_‐type ordered phase (space group *Pm*3*m*). As illustrated in Figure 1a, within the unit cell, Mn atoms occupy the face‐centered positions, while Pt atoms are located at the cube corners, forming a highly symmetric crystal framework. A key structural feature is the presence of Kagome‐like layers of Mn atoms parallel to the (111) planes. Within these layers, the Mn moments are arranged in a triangular 120° spin configuration, giving rise to the nc‐AFM order characteristic of this material. Epitaxial Mn*_x_* Pt thin films (25 nm) are deposited on MgO (001) substrates via co‐sputtering from elemental Mn and Pt targets. The stoichiometry *x* of the films is precisely controlled by tuning the sputtering power of the Mn target. The corresponding device stack, illustrated in Figure 1b, consists of MgO (001) substrate / Mn_3_Pt (25 nm) / Al_2_O_3_ (100 nm) / Ta (15 nm). Here, a 100 nm‐thick Al_2_O_3_ layer deposited by atomic layer deposition (ALD) serves as an insulating thermal barrier to establish a controlled out‐of‐plane temperature gradient, while a 15 nm‐thick Ta layer is sputtered on top as a resistive heating strip. This structure simultaneously ensures the single‐crystal quality of the Mn_3_Pt film and fulfills the requirements for generating and measuring the thermal gradient (∇*T*) in thermoelectric characterization. The single‐crystal quality and epitaxial nature of the Mn_3_Pt films are confirmed by x‐ray diffraction (XRD). The out‐of‐plane *θ*‐2*θ* scan (Figure 1c) shows distinct (001) and (002) peaks of Mn_3_Pt alongside the MgO substrate peaks, indicating high‐quality (001)‐oriented growth. XRD patterns of Mn*_x_* Pt films with other Mn compositions are presented in Figure S1a. Epitaxial alignment is further verified by *φ*‐scans of the Mn_3_Pt (111) and MgO (111) families of planes (Figure 1d and Figure S1b), which exhibit a fourfold symmetry with coincident peaks, confirming a cube‐on‐cube epitaxial relationship. This finding is further corroborated by the polar coordinate plot derived from the Mn_3_Pt (111) reflection (Figure 1e). Atomic Force Microscopy (AFM) characterizations confirm that all Mn*_x_* Pt films exhibit smooth surfaces with low roughness average (Ra) roughness (Figure S2). Additionally, the exceptional crystalline quality of the film is unambiguously demonstrated by atomic‐resolution high‐angle annular dark‐field (HAADF) imaging (Figure S3).

**FIGURE 2:**
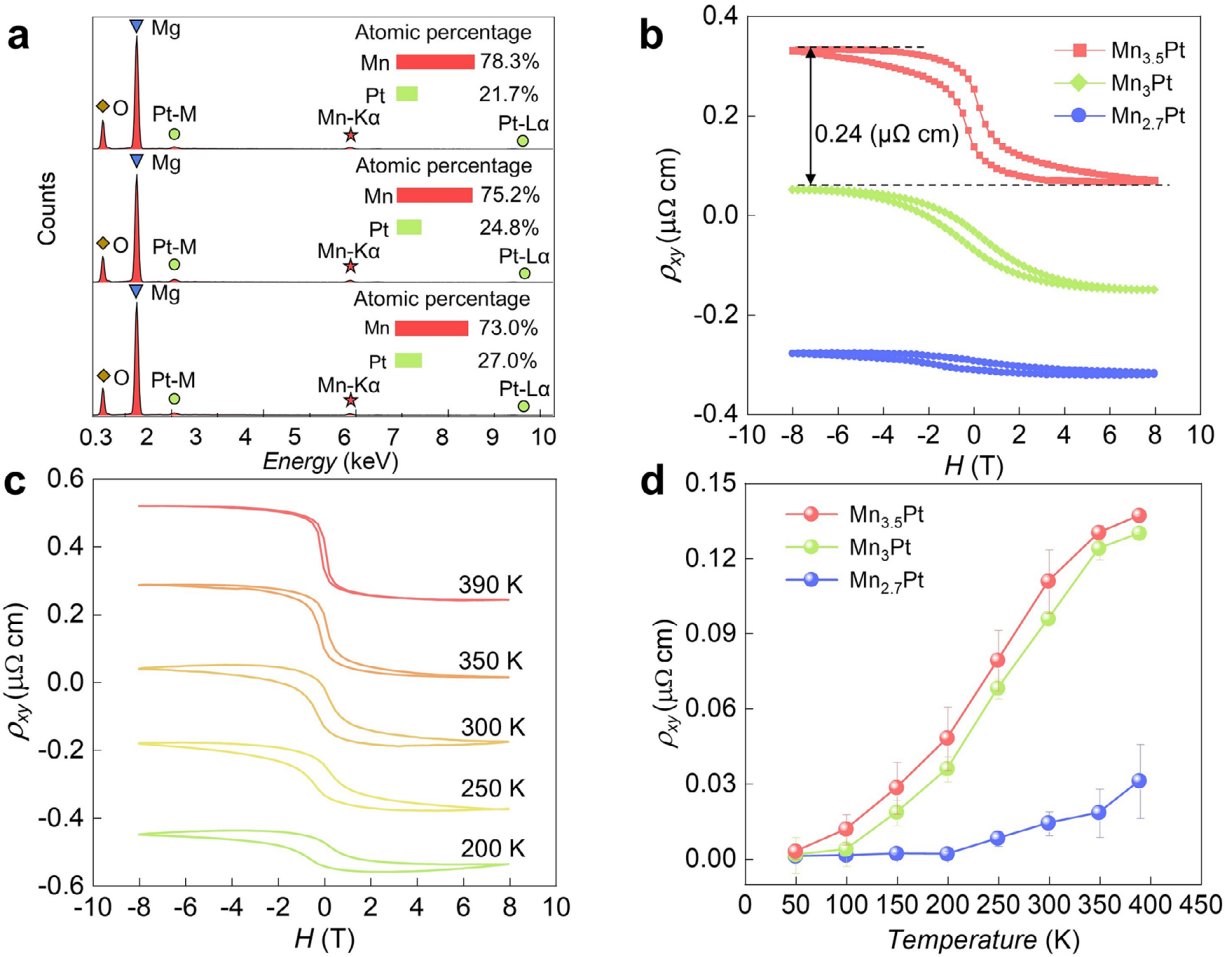
(a) Representative EDS spectrum for films with varying *x*. (b) AHE loops for *x* = 2.7, 3.0, and 3.5 at 300 K after subtracting the ordinary Hall effect contribution. (c) AHE loops of Mn_3.5_Pt at different temperatures. (d) Temperature dependence of ρ_*xy*_.

For compositional modulation, a series of Mn*_x_* Pt films grown under different conditions is characterized by energy‐dispersive spectroscopy (EDS). As shown in Figure 2a, the Mn stoichiometry (*x*) in Mn*_x_* Pt is continuously tuned from 2.7 to 3.5 by adjusting the Mn sputtering power (70–90 W) during the co‐sputtering process. To ensure the accuracy of the EDS composition analysis, measurements are performed on multiple regions of each sample with varying acceleration voltages (Figures S4 and S5). This precise stoichiometric control establishes a crucial foundation for systematically investigating the composition‐dependent electromagnetic and thermoelectric properties. As shown in Figure S6, the in‐plane magnetization measurements for the three compositions all exhibit low saturation magnetizations (∼13 emu/cc), consistent with the nc‐AFM phase of Mn_3_Pt. The AHE for Mn*_x_* Pt films is shown in Figure 2b, measured with the magnetic field parallel to the *z*‐axis at 300 K. The temperature dependence is shown in Figure S7. At a fixed temperature, the zero‐field anomalous Hall resistivity $\rho_{xy_{0}}$ (ρ_*xy*_ at H = 0 T) increases monotonically with Mn content, indicating an enhanced intrinsic Berry curvature originating from the noncollinear spin texture. This systematic variation is driven by alterations in the electronic structure upon Mn doping. Elevating the Mn concentration introduces additional local magnetic moments and perturbs the electronic states, resulting in significant band renormalization. This renormalization yields increased band overlap and a diminished band gap around the Fermi surface, an electronic environment highly conducive to the amplification of the Berry curvature near specific k‐points in the Brillouin zone. On the other hand, the mechanism linking the observations lies in the origin of the Berry curvature. It is a geometric property of the electronic wavefunctions in momentum space, which in nc‐AFMs is induced by spin–orbit coupling (SOC) and the noncollinear magnetic order. Enhanced magnetic exchange interactions provide the key to stabilizing this magnetic order, which subsequently shapes the topological band structure and ultimately governs the magnitude of the Berry curvature. Therefore, an increase in Mn content is expected to modify the interatomic magnetic exchange interactions. In our case, the observed enhancement is consistent with a scenario in which this modification effectively stabilizes the noncollinear spin texture, thereby enhancing the Berry curvature and leading to the increased AHE. The temperature dependence of the enhanced AHE in Mn*_x_* Pt is further investigated. As shown in Figure 2c, the anomalous Hall resistivity (ρ_*xy*_) of Mn_3.5_Pt increases notably with temperature. This quantity is defined as:

(1) $$\rho_{xy}=\frac{E_{xy}}{J_{xx}}=\frac{V_{xy}/d}{I_{xx}/\left(d\cdot t\right)}=R_{xy}\cdot t$$

here, *E_xy_* denotes the transverse electric field generated along the width of the Hall bar, and *J_xx_* denotes the longitudinal current density. In the expression, ν_*xy*_ represents the transverse voltage measured across the Hall bar, *d* is the width of the Hall bar, *t* is the thickness of the film, and *I_xx_* is the longitudinal current. The Hall resistance is defined as  *R_xy_* = ν_*xy*_/*I_xx_*. Therefore, the Hall resistivity ρ_*xy*_ is given by the product of the Hall resistance and the film thickness. The ρ_*xy*_ of Mn*_x_* Pt increases with temperature, as shown in Figure 2d. This temperature dependence originates from the intensification of lattice vibrations at elevated temperatures, which disrupts the noncollinear magnetic order and reduces the net magnetization. The AHE conductivity, being dominated by the Berry phase contribution, is proportional to the net magnetization. Therefore, the reduction in AHE conductivity, driven by the weakening magnetization, directly results in the observed increase of the $\rho_{xy_{0}}$.

**FIGURE 3:**
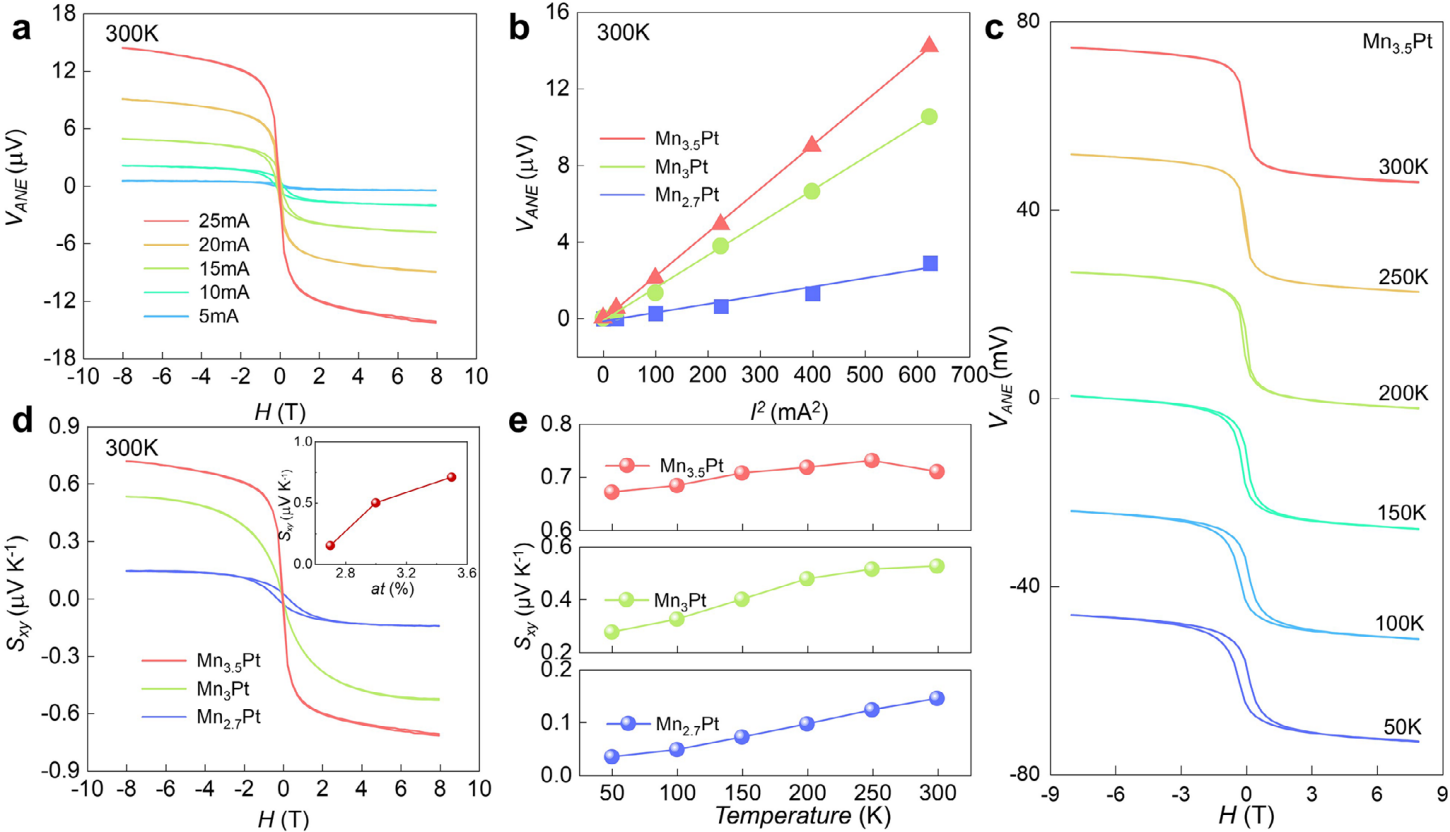
(a) ANE loops for Mn_3.5_Pt at 300 K with different heating currents. (b) Linear relationship between *V_ANE_* and the square of the current (*I* ^2^) at 300 K. (c) ANE loops of Mn_3.5_Pt at different temperatures. The heating current is 25 mA. (d) Field dependence of the anomalous Nernst coefficient *S_xy_* for Mn*_x_* Pt films (*x* = 2.7, 3.0, 3.5) at 300 K. The inset plots the *S_xy_* versus the Mn/Pt atomic ratio. The heating current is 25 mA. (e) Temperature dependence of *S_xy_* for Mn*_x_* Pt.

Furthermore, Mn doping effectively modulates the ANE in Mn*_x_* Pt. This tunability stems from the shared origin of the ANE and the AHE in the Berry curvature of the electronic bands. The two effects are distinguished by their respective response functions. The AHE is governed by the Berry curvature summed over all occupied states up to the Fermi energy, making it sensitive to the Fermi‐surface geometry (Equation 2). In contrast, the ANE is proportional to the Berry curvature moment, which involves an energy derivative and is therefore highly sensitive to variations in the density of states and the energy‐dependent Berry curvature near the E_*F*_ (Equation 3).

(2) $$\sigma_{xy}^{AHE}=\frac{j_{y}}{E_{x}}=-\frac{e^{2}}{h}\int \frac{d^{3}k}{{\left(2\pi \right)}^{3}}\Omega_{z}\left(k\right)f\left(k\right)$$

(3) $$\alpha_{xy}^{ANE}=-\frac{1}{eT}\int \frac{d^{3}k}{{\left(2\pi \right)}^{3}}\Omega_{z}\left(k\right)\left(-\frac{\partial f}{\partial \in }\right)v_{x}\left(k\right)\tau \left(k\right)$$

here, *j_y_* is the transverse current density generated in the *y*‐direction, and *E_x_* is the longitudinal electric field applied in the *x*‐direction. The *e* denotes the elementary charge, and ℏ is the reduced Planck constant. The integration is carried out over the three‐dimensional Brillouin zone, where *d* ^3^ *k* is the volume element in momentum space and (2π)^3^ is the normalization factor. The quantity Ω_*z*_(**k**) is the *z*‐component of the Berry curvature at wavevector **k**, and *f*(**k**) is the Fermi–Dirac distribution function. Moreover, *T* is the absolute temperature. The factor $\left(-\frac{\partial f}{\partial \in }\right)$ is the energy derivative of the Fermi–Dirac distribution, which restricts the dominant contribution to electronic states near the Fermi surface. The quantity *v_x_* (**k**) is the *x*‐component of the electron group velocity, and τ(**k**) is the relaxation time. As illustrated in Figure 1b, a vertical ∇*T* is generated normal to the film plane by applying an alternating current to a Ta strip heater. The detailed calibration procedure is described in Figure S8. The ANE voltage (*V_ANE_*) is measured in the direction perpendicular to both the applied in‐plane magnetic field and the ∇*T*. For Mn_3.5_Pt, the measured ANE voltage shows a perfect quadratic dependence on the applied heating current, confirming the purely Joule origin of the ∇*T* (Figure 3a). Measurements on films with different stoichiometric ratios (Figure 3b) exhibit the same quadratic dependence, confirming that this behavior is consistent across the Mn*_x_* Pt system. Notably, as shown in Figure 3c, Mn_3.5_Pt exhibits no significant variation in its *V_ANE_* with the ambient temperature, demonstrating excellent thermal stability. Furthermore, Figure 3d presents the magnetic field dependence of the ANE coefficient for Mn*_x_* Pt (*S_xy_*) at 300 K, which is calculated as:

(4) $$S_{xy}=\frac{V_{ANE}}{\nabla T}\cdot \frac{L_{z}}{L_{x}}$$

where *V_ANE_* is the ANE voltage, ∇*T* is the vertical thermal gradient, and *L_z_* and *L_x_* represent the distances for measuring the ∇*T* and the *V_ANE_*, respectively. The *S_xy_* exhibits a significant dependence on *x*, a trend that correlates with the composition‐dependent saturation of the $\rho_{xy_{0}}$. Furthermore, *S_xy_* demonstrates a characteristic temperature evolution across the different samples, as shown in Figure 3e (detailed data are shown in Figures S9,S10). The gradual increase of *S_xy_* with temperature is associated with a magnetic phase transition in Mn*_x_* Pt. At low temperatures, the system resides in a spin‐glass state. Upon warming, the magnetic configuration transitions from this disordered state to a nc‐AFM ordering, leading to the enhancement of the Berry‐curvature‐driven ANE. Notably, for Mn_3.5_Pt, the *S_xy_* reaches a value as high as 0.71 µV/K. This value remarkably exceeds the room‐temperature *S_ij_* (0.4 µV/K) reported for the well‐known nc‐AFMs of Mn_3_Sn and Mn_3_Ge.

**FIGURE 4:**
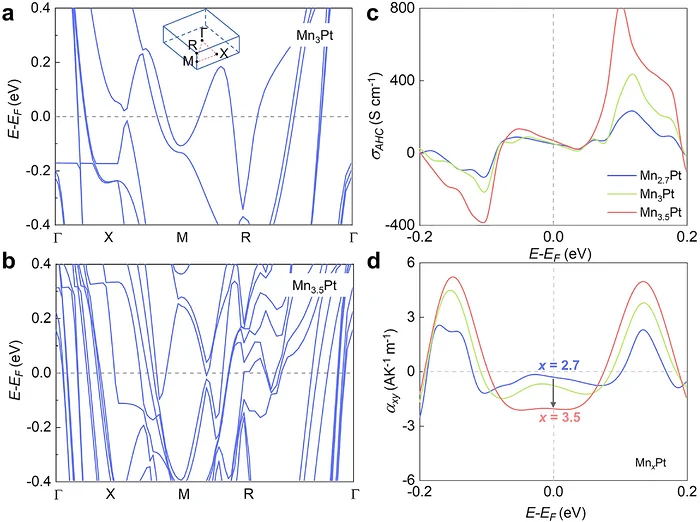
DFT‐calculated band structures of (a) Mn_3_Pt and (b) Mn_3.5_Pt. The gray dashed line marks the E_*F*_. (c) Energy dependence of the σ_*AHC*_. (d) Energy dependence of α_*xy*_ for different compositions.

To elucidate the electronic origin of the observed ANE, first‐principles calculations based on density functional theory (DFT) are performed for Mn_3_Pt. As shown in Figure S11, we constructed 2 × 2 × 2 supercells based on the ordered L1_2_ structure of Mn_3_Pt to model the non‐stoichiometric Mn_2.7_Pt and Mn_3.5_Pt compositions. Figure S12a presents the Berry‐curvature‐weighted band structure of Mn_3_Pt from DFT calculations. Band projection analysis reveals that the electronic bands within ± 2 eV of the E_*F*_ are dominated by the Mn‐3d orbitals, with a minor contribution from the Pt‐5d states. The strong *d*‐*p* orbital hybridization of Mn‐3*d* electrons and spin–orbit coupling jointly break the time‐reversal symmetry, driving the system to form a noncollinear magnetic order (based on the Dzyaloshinskii–Moriya interaction). This establishes the electronic structure foundation for the generation of Berry curvature and the occurrence of the ANE. The band structures of Mn_3_Pt and Mn_3.5_Pt are presented in Figure 4a,b, respectively. Consistent with this compositional control, the incorporation of additional Mn atoms breaks the original crystal symmetry of Mn_3_Pt. From a band structure perspective, this symmetry breaking lifts degeneracies and induces additional splitting of the previously overlapping bands. The resulting repulsion and separation of these sub‐bands enhance the topological nontriviality of the electronic states in momentum space, which significantly amplifies the overall Berry curvature. This amplification is directly manifested as the observed increase in the AHE signal. As shown in Figure 4c, although the observed increase in the magnitude of anomalous Hall conductivity (σ_*AHC*_) is relatively limited, a remarkably significant increase in the slope of σ_*AHC*_ occurs within the energy range near the E_*F*_. According to the Mott relation (Equation 5), this change ($\frac{d\sigma_{ij}}{dE}$) directly results in a substantial growth of the anomalous Nernst conductivity (α_*ij*_). This is well in line with the expected results of our experiment.

(5) $$\alpha_{ij}=\frac{\pi^{2}}{3}\;\frac{k_{B}^{2}T}{e}\;\frac{d\sigma_{ij}}{dE}$$

In addition, Figure S7b unveils that the large intrinsic Berry curvature in Mn_3_Pt stems from its distinctive Fermi surface topology, which comprises multiple nested closed pockets and hosts pronounced Berry curvature hotspots (the peak value of Berry curvature can reach the order of 10^2^ Å) near topological singularities like Weyl points. This discovery thereby establishes Mn_3_Pt as an ideal platform for investigating the ANE. The calculated anomalous Nernst conductivity α_*xy*_ of the Mn*_x_* Pt system as a function of energy is presented in Figure 4d, with the E_*F*_ indicated by the gray dashed line. The data shows that as the Mn increases, the α_*xy*_ of the E_*F*_ exhibits a monotonically increasing trend. Notably, a value of 2.06 AK^−1^m^−1^ is achieved for Mn_3.5_Pt. These computational results robustly confirm the decisive influence of Berry curvature on the ANE in the Mn*_x_* Pt system, providing direct evidence for the concomitant enhancement of the *S_xy_* with increasing *x*.

**FIGURE 5:**
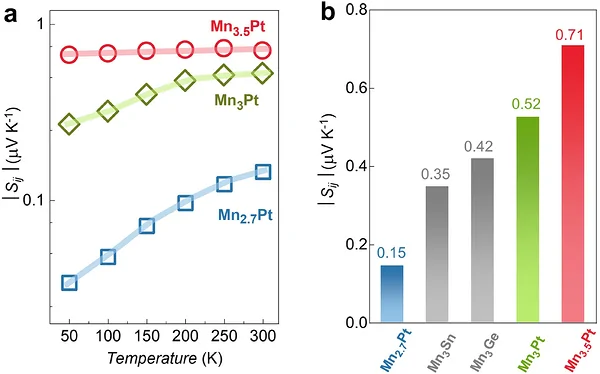
(a) A comparative overview of the temperature‐dependent *S_ij_* across different Mn*_x_* Pt. (b) The *S_ij_* for the typical Mn_3_X at 300K.

The temperature‐dependent *S_ij_* for various Mn*_x_* Pt under standard conditions are plotted in Figure 5a. Moreover, Figure 5b presents a comparison of the room‐temperature (300 K) *S_ij_* values obtained for the Mn*_x_* Pt samples in this work with those of typical Mn_3_X (X = Sn, Ge) compounds. Compared to other nc‐AFMs, Mn_3.5_Pt demonstrates highly efficient thermoelectric conversion performance at room temperature. Notably, Mn_3.5_Pt maintains the performance stably within the temperature range of 50–300 K, with a performance fluctuation of less than 10%. This thermal robustness suggests a competition between the intrinsic topological nature of the Berry curvature and the statistical behavior of electron scattering. Microscopically, the transverse thermoelectric conductivity of ANE is dominated by the integral of the Berry curvature of electrons near the *E_F_*. As a topological quantity describing the geometric phase of electron wavefunctions, the Berry curvature exhibits an intrinsic distribution in momentum space. For topologically nontrivial Mn_3_X series compounds, the strong spin–orbit coupling and magnetic ordering of Mn‐3d electrons at E_*F*_ induce localized topological peak structures of the Berry curvature at the Fermi surface. This intrinsic topological contribution, being temperature‐insensitive, serves as an “anchor” to maintain the stability of |*S_ij_* |. Meanwhile, the evolution of electron scattering processes with temperature constitutes the other competing party. In conventional transport, the probability of electron‐phonon and electron‐impurity scattering increases with rising temperature, typically leading to a monotonic decay of transport coefficients. However, in systems like Mn_3.5_Pt, the topological enhancement effect of Berry curvature and the attenuation effect of scattering precisely reach a dynamic equilibrium. At low temperatures, although scattering is weak, the topological contribution of Berry curvature is not fully prominent due to the screening of electronic states by the Fermi surface. As temperature increases, the ANE in Mn_3.5_Pt exhibits exceptional robustness against this scattering due to the localized Berry curvature near *E_F_*. The competition eventually reaches a delicate balance, resulting in the nearly constant |*S_ij_* | over a wide temperature range. To verify this competition picture, we investigate the ρ_*xy*_ as a function of ρ_*xx*_ with varying temperatures in all three samples (see Figure S13). After calculating the intrinsic contribution from Berry curvature and extrinsic contribution from scattering, we found the Berry curvature contribution in Mn_3.5_Pt is most dominant in all three samples, aligning well with our assumption, as the giant and temperature‐invariant ANE in Mn_3.5_Pt is dominated by the intrinsic Berry curvature rather than scattering effects. The temperature‐dependent curves of other Mn‐based compounds (Mn_3_Sn) further corroborate the universality of this competitive mechanism. The slowly increasing behavior of Mn_3_Sn reflects a dynamically adjusting competition between Berry curvature and scattering. In contrast, the plateau feature of Mn_3.5_Pt signifies that the topological intrinsic nature of Berry curvature completely suppresses the statistical nature of scattering, becoming the dominant factor in transport. Therefore, the strong performance stability of Mn_3.5_Pt demonstrates its significant potential for applications in complex environments, making it a promising candidate for thermoelectric devices.

## 3 Conclusion

In summary, we have demonstrated that the nc‐AFM Mn_3_Pt, in its high‐quality thin‐film form, exhibits a giant and remarkably temperature‐invariant ANE. Crucially, by employing Mn doping, we have successfully mitigated the fundamental challenge posed by the high crystal symmetry in cubic antiferromagnets. Through precise stoichiometric control in Mn_3_Pt films, we achieved a systematic enhancement of both the AHE and the ANE. This tunability is directly linked to the amplification of the intrinsic Berry curvature near the *E_F_*, as confirmed by our first‐principles calculations, which show that Mn doping modifies the electronic structure and enhances the topological nontriviality.

The culmination of this optimization is the Mn_3.5_Pt composition, which showcases a superior room‐temperature *S_ij_* of 0.71 µV/K, surpassing other prominent nc‐AFMs like Mn_3_Sn and Mn_3_Ge. More importantly, this performance remains exceptionally stable over a broad temperature range (50–300 K), a feature we attribute to a finely tuned dynamic equilibrium between the robust, temperature‐insensitive Berry curvature and conventional electron scattering processes. This combination of a large response and outstanding thermal stability overcomes a critical limitation in thermoelectric. Our work establishes Mn_3_Pt thin films as a premier material platform for antiferromagnetic spintronics and micro‐thermoelectric energy harvesting. The giant, stable ANE signal, coupled with a substantial coercive field and the inherent advantages of antiferromagnets, positions Mn_3_Pt as an ideal candidate for developing efficient, field‐free, and broadband thermal energy conversion devices for next‐generation electronics.

## 4 Materials and Methods

### 4.1 Film Growth and Crystalline Characterization

Mn_3_Pt thin films are epitaxially grown on single‐crystal MgO (001) substrates via magnetron sputtering. The deposition was performed at 550°C in an argon atmosphere maintained at 2.6 mTorr. Mn and Pt are co‐sputtered using DC (power: 70, 80, and 90 W) and RF (power: 30 W) targets, respectively. Subsequently, a post‐growth annealing is conducted at 600°C for 1 h in high vacuum. The crystal structure of the films is characterized by Bruker x‐ray diffraction with a Cu K𝛼 source.

### 4.2 Magnetization and Magneto‐Thermal Transport Measurements

Hall bar devices are patterned using standard photolithography and argon ion etching (TuoTuo Technology, UV Litho‐ACA) (Figure S8b). A 100 nm‐thick Al_2_O_3_ layer is then deposited uniformly over the device via ALD. A 15 nm‐thick Ta thin film is subsequently grown on the surface of the Al_2_O_3_ layer to serve as an on‐chip heater for generating a vertical temperature gradient. The heating strip is shaped as an I‐beam with a width of 20 µm. Both the AHE and ANE are characterized from 50 to 390 K using a Physical Property Measurement System (PPMS, Quantum Design).

### 4.3 The First Principles Calculation

First‐principles calculations are performed within the framework of DFT as implemented in the Quantum ESPRESSO package [61]. The Perdew–Burke–Ernzerhof (PBE) generalized gradient approximation is adopted for the exchange‐correlation potential. A plane‐wave kinetic energy cutoff of 500 eV and a 7 × 7 × 7 k‐point mesh are used for self‐consistent calculations. The convergence criteria are set to 10^−6^ eV for total energy and 0.001 eV/Å for atomic forces during geometric optimization. Tight‐binding Hamiltonians constructed using the WANNIER90 package [62]. The anomalous Nernst conductivity, Fermi surface, and Berry curvature are then calculated from the Wannier‐interpolated tight‐binding Hamiltonian using the WANNIERTOOLS package [63].

## Author Contributions

X.P.Q. and R.Q.C. conceived and supervised the project. P.W.G., W.Z., M.Z.W., Y.H.L., and C.P. prepared the materials and performed the material characterizations, device fabrication, and electrical transmission measurement. A.Q.X, Y.G.D., and J.X.C. contributed to the material characterizations. Y.C.Z. contributed to the device fabrication. X.L.Z. performed the theoretical calculations. P.W.G., X.L.Z., Z.S., D.F.S., L.L, S.M.Z., Y.C.G., R.Q.C., and X.P.Q. analyzed the data and co‐wrote the manuscript in consultation with all the other authors.

## Conflicts of Interest

The authors declare no competing interests.

## Supporting information




**Supporting File**: advs75880‐sup‐0001‐SuppMat.docx.

## Data Availability

The data that support the findings of this study are available on request from the corresponding author. The data are not publicly available due to privacy or ethical restrictions.
