# Supplementary material for: Large Temperature‐invariant Anomalous Nernst Effect in Non‐collinear Antiferromagnet Mn3Pt
Source: Adv Sci (Weinh). 2026 May 29;13(47):e75880. doi: 10.1002/advs.75880 (PMC13335787; doi:10.1002/advs.75880)
Supplement: Supplementary file 1 [file ADVS-13-e75880-s001.docx]

**Large Temperature-invariant Anomalous Nernst Effect in Non-collinear Antiferromagnet Mn_3_Pt**

Pengwei Gong^1†^, Xiaolin Zhang^2†^, Wei Zhu^1^, Mingzhi Wang^1^, Chang Pan^1^, Aoqi Xu^1^, Yandong Guo^1^, Yuheng Li^1^, Jiaxin Chen^3^, Yanchang Zhou^2^, Zhong Shi^1^, Dingfu Shao^4^, Liang Liu^3^, Shiming Zhou^1^, Yicheng Guan^1*^, Ruiqing Cheng^2*^, Xuepeng Qiu^1^^*^

*^1^School of Physics Science and Engineering, Tongji University, Shanghai 200092, China*

*^2^School of Physics and Technology, Wuhan University, Wuhan 430072, China*

*^3^Tsung-Dao Lee Institute, Shanghai Jiao Tong University, Shanghai 201210, China*

*^4^Key Laboratory of Materials Physics, Institute of Solid State Physics, Hefei Institutes of Physical Science, Chinese Academy of Sciences, Hefei 230031, China*

**^†^**These authors contributed equally to this work.

^*^Email: ycguan@tongji.edu.cn, chengrq@whu.edu.cn, xpqiu@tongji.edu.cn

**FIGURE S1** (**a**) Out-of-plane *θ-2θ* XRD scan measurements of Mn_2.7_Pt, Mn_3_Pt, and Mn_3.5_Pt. (**b**) *φ*-scans around the (111) of Mn_2.7_Pt, Mn_3_Pt, and Mn_3.5_Pt.

Mn_3_Pt thin films are fabricated by magnetron co-sputtering with controlled Mn sputtering power, yielding three samples with distinct Mn: Pt stoichiometry ratios (2.7, 3.0, and 3.5). Figure S1a presents the XRD scans, in which all films exhibit clear (001) and (002) diffraction peaks of the Mn_3_Pt phase, confirming its successful formation. Variations in peak intensity and shape further indicate composition-dependent structural evolution. Figure S1b shows the φ-scan profiles. The Mn_3_Pt (111) peaks for each film align with the MgO (111) substrate peaks with a 90° periodicity, confirming epitaxial growth on MgO (001). Notably, even the non-stoichiometric Mn_2.7_Pt and Mn_3.5_Pt films maintain epitaxy, though peak broadening suggests an increase in structural disorder. These results demonstrate that co-sputtering enables tunable composition control and high-quality epitaxial growth of Mn_3_Pt thin films.

**FIGURE S2** Atomic force microscopy (AFM) topography images of (**a**) Mn_2.7_Pt, (**b**) Mn_3_Pt, and (**c**) Mn_3.5_Pt thin films, respectively.

As shown in Figure S2a-2c, the AFM images (5μm×5μm) reveal that all films exhibit smooth surfaces with small roughness average (Ra) roughness, 0.59 nm, 0.56 nm and 0.57 nm for the Mn_2.7_Pt, Mn_3_Pt, and Mn_3.5_Pt films, respectively.

**FIGURE S3** Atomic-resolution high-angle annular bright-field (HAABF) image of (001) Mn_3_Pt thin film**,** shown at scale bars of (**a**) 5 nm and (**b**)2 nm.

Figure S3a shows a Scanning Transmission Electron Microscopy (STEM) image of a Mn_3_Pt thin film which scale bar is 5 nm, revealing the overall surface morphology of the film. A magnified STEM view resolves the atomic structure of the Mn_3_Pt (001) plane, where ordered Pt and Mn atomic sites are clearly distinguished along the (-1-10) crystallographic direction in Figure S3b. These observations directly confirm the well-ordered crystalline arrangement of the Mn_3_Pt film.

**FIGURE S4** (**a**) Schematic illustration of three distinct regions (labeled 1, 2, and 3) on a 5 mm × 5 mm Mn*_x_*Pt film where EDS measurements are performed. (**b**) Mn/Pt atomic ratios measured by EDS for Mn_2.7_Pt, Mn_3_Pt, and Mn_3.5_Pt films across the three regions. (**c**) Optical micrograph showing six individual Hall bar devices patterned at different positions on the same film. (**d**) $V_{\text{ANE}}$ measured from four representative devices (labeled 1-4) under identical conditions ($T=300$ K, heating current $I=15$ mA).

The compositional uniformity of the Mn*_x_*Pt thin films is confirmed by multiple complementary measurements. First, EDS analysis performed on three distinct regions of each 5 mm × 5 mm film (Figure S4a-4b) reveals nearly identical Mn/Pt stoichiometry ratios in all three samples of Mn_2.7_Pt, Mn_3_Pt, and Mn_3.5_Pt, with variations within ± 0.3%, demonstrating excellent compositional homogeneity. Moreover, six Hall bar devices patterned at different positions on the same film (Figure S4c) show highly consistent $V_{\text{ANE}}$ under identical conditions ($T=300$ K, $I=15$ mA), as shown in Figure S4d. Notably, Device 2, which is used for the main measurements in this study, exhibits nearly identical $V_{\text{ANE}}$ to the other three devices. Given the extreme sensitivity of the ANE to the Fermi level position, this consistent electrical response across multiple devices provides strong evidence that the film thickness, interface quality, and stoichiometry are uniform across the entire sample.

**FIGURE S5 (a)** The reference CoPt (20 nm) thin film measured at different accelerating voltages (15 kV, 20 kV, and 25 kV). Mn/Pt atomic ratios of (**b**) Mn_2.7_Pt, (**c**) Mn_3_Pt, and (**d**) Mn_3.5_Pt thin films, measured under the different accelerating voltage conditions.

 The reference CoPt (20 nm) thin film measured at different accelerating voltages, showing excellent agreement with the nominal equiatomic composition (Co_50_Pt_50_) in Figure S5a. Additionally, to evaluate the influence of substrate penetration on quantification, we performed EDS measurements at multiple accelerating voltages-specifically 15 kV (the condition used in the manuscript), 20 kV, and 25 kV on all three Mn*_x_*Pt compositions. Remarkably, the measured Mn/Pt atomic ratios in Figure S5b-5d remain nearly constant across this voltage range for each sample, with variations within ±0.3%. This voltage-independent behavior provides strong evidence that the quantification is robust within this excitation window.

**FIGURE S6** Magnetization as a function of in-plane applied magnetic field measured at 300 K for Mn_2.7_Pt, Mn_3_Pt, and Mn_3.5_Pt.

As compiled in Figure S6, in-plane magnetization at 300 K for all three samples yields saturation magnetization values of approximately 13 emu/cc, which is in excellent agreement with the reported value for nc-AFM Mn3Pt single crystals (~15 emu/cc) and two orders of magnitude smaller than that of ferromagnetic MnPt_3_. This low magnetization cannot arise from ferromagnetic or strong ferrimagnetic order and is fully consistent with the weak residual magnetization from slight canting of the triangular spin structure in nc-AFM Mn_3_Pt.

**FIGURE S7** Field dependence of the AHE of (**a**) Mn_2.7_Pt, (**b**) Mn_3_Pt, and (**c**) Mn_3.5_Pt from 50 K to 390 K.

Figure S7 shows the temperature-dependent AHE in Mn_3_Pt thin films with three different Mn: Pt atomic ratios (2.7, 3.0, and 3.5). The $V_{\text{AHE}}$ is plotted against the applied magnetic field $H$ from 50 K to 390 K.

**FIGURE S8** (**a**) Schematic of the device geometry. (**b**) Optical micrograph of the device. (**c**) Resistance response of the Ta strip under sensing current (0.01 mA) and heating current (25 mA). (**d**) The determination of the temperature difference $\Delta T=T_{2}-T_{1}$ across the film.

The device geometry and measurement parameters are presented in Figures S8a-8b. The temperature gradient across the film is determined using the differential temperature method. Specifically, we using the Ta strip as both a heater and a resistance thermometer. The sample is first mounted in a Physical Property Measurement System (PPMS) to establish a stable base temperature, which is taken as the bottom temperature $T_{1}$ of the film. Firstly, the Ta strip is calibrated as a resistance thermometer by measuring its resistance $R_{Ta}$ as a function of $T_{1}$ with a sensing current of 0.01 mA, thereby yielding a linear calibration curve, $R_{Ta}(T)$. Subsequently, a heating current of 25 mA is applied, with the resulting resistance recorded as $R_{\text{Ta}}^{\text{heating}}$. The temperature-dependent resistance of the Ta strip and the temperature gradient extracted from the linear fit are shown in Figures S8c-8d. Since the resistance of the Ta strip depends solely on its own temperature, regardless of whether the heating originates from the PPMS environment or Joule heating, the calibration curve is used to convert $R_{\text{Ta}}^{\text{heating}}$ into the actual temperature at the top surface of the film, denoted as $T_{2}$. The temperature difference across the film is then obtained as $\Delta T=T_{2}-T_{1}$.

**FIGURE S9** Magnetic field dependence of *V_ANE_* for (**a**) Mn_2.7_Pt, (**b**) Mn_3_Pt, and (**c**) Mn_3.5_Pt from 50K to 300K.

The Figure S9 presents the temperature-dependent anomalous Nernst effect (ANE) of Mn_3_Pt thin films with three different Mn: Pt stoichiometric ratios (2.7, 3.0, and 3.5). The anomalous Nernst voltage ($V_{\text{ANE}}$) is plotted as a function of the applied magnetic field $H$ over the temperature range of 50-300 K.

**FIGURE S10** Magnetic hysteresis loop of *V_ANE_* with different applied currents *I* in (**a**) Mn_2.7_Pt, (**b**) Mn_3_Pt, and (**c**) Mn_3.5_Pt film at 300 K.

Figures S10a-10c present $V_{\text{ANE}}$ as a function for Mn*_x_*Pt films with Mn: Pt stoichiometric ratios of 2.7, 3.0, and 3.5, under bias currents ranging from 5 mA to 25 mA. The magnitude of $V_{\text{ANE}}$ increases gradually with rising current. This trend is consistent with enhanced Joule heating, which generates a larger temperature gradient-the primary driving force of the ANE.

**FIGURE S11** (**a**) 2×2×2 supercell of stoichiometric Mn_3_Pt with the ordered L1_2_ structure. (**b**) Computational model for Mn_3.5_Pt. (**c**) Computational model for Mn_2.7_Pt.

Specifically, to model the non-stoichiometric compositions Mn_2.7_Pt and Mn_3.5_Pt, we constructed 2×2×2 supercells based on the ordered L1_2_ structure of Mn_3_Pt as shown in Figure S11a, which contains 32 atoms (24 Mn and 8 Pt) in the unit cell. For Mn_3.5_Pt, we introduced an additional Mn atom by replacing one Pt atom with an Mn atom in the supercell (Figure S11b). This gives a supercell composition of Mn_25_Pt_7_, corresponding to an Mn: Pt ratio of 3.6:1, which is close to the target Mn3.5 composition. For Mn_2.7_Pt, we replaced one Mn atom with a Pt atom in the supercell, resulting in a composition of Mn_23_Pt_9_, corresponding to an Mn: Pt ratio of 2.6:1, which is close to the target Mn2.7 composition (Figure S11c).

**FIGURE S12** (**a**) DFT-calculated weight band structure of Mn_3_Pt. Pink, yellow, and orange solid dots represent the electron contributions from the s, p, and d orbitals of the Mn atom, respectively; Light blue, dark blue, and purple solid dots represent the electron contributions from the s, p, and d orbitals of the Pt atom, respectively. (**b**) Fermi surface and Berry curvature maps in the *k*_z_ = 0 plane of the Brillouin zone.

The Figure S12a presents the orbital-projected band structure of Mn_3_Pt obtained from first-principles calculations, with the Fermi level ($E_{F}$) set as the dashed reference line. Notably, Mn-$d$ orbitals (orange) dominate the electronic structure over a broad energy range, particularly near $E_{F}$. Their pronounced spectral weight in this region indicates that Mn-$d$ states are the primary contributors to the Fermi‑level electronic states, which is crucial for determining the magnetic and transport properties of Mn_3_Pt. Pt‑$d$ orbitals (purple) also make a non‑negligible contribution, reflecting significant Mn‑$d$/Pt‑$d$ orbital hybridization that further modulates the electronic structure. In contrast, Mn‑$s/p$ and Pt‑$s/p$ orbitals show only weak spectral weight, confirming their minor role in the dominant electronic states near the Fermi level. These orbital-projected results demonstrate that the key physical properties of Mn_3_Pt originate predominantly from its Mn‑$d$ electronic states. Figure S12b displays the Berry-curvature distribution (color scale) superimposed on the Fermi surface (black contours) of Mn_3_Pt. Pronounced extrema in the Berry curvature-seen as high‑magnitude purple regions-coincide with topological features of the Fermi surface.

**FIGURE S13** Anomalous Hall resistivity $\rho_{xy}$ plotted against longitudinal resistivity $\rho_{xx}$ for (**a**) Mn_2.7_Pt, (**b**) Mn_3_Pt, and (**c**) Mn_3.5_Pt over the temperature range of 50 K to 300 K. (**d**) Comparison of the intrinsic-to-extrinsic ratio ${\mid b}_{in}/a_{sk}\mid$ for the Mn_2.7_Pt, Mn_3_Pt, and Mn_3.5_Pt. (**e**) Temperature- dependent $\rho_{xx}$ and (**f**) $\rho_{xy}$ for the Mn_2.7_Pt, Mn_3_Pt, and Mn_3.5_Pt.

We systematically measured the temperature-dependent longitudinal resistivity $\rho_{xx}(T)$ from 50 K to 350 K for all three Mn*_x_*Pt compositions ($x=$ 2.7, 3.0, and 3.5), with results shown in Figure S13a-S13c. We further performed the AHE scaling analysis: $\rho_{xy}=a_{sk}\rho_{xx}+b_{in}\rho_{xx}^{2}$, where the first term represents the skew scattering contribution and the second term describes the intrinsic AHE arising from time-reversal symmetry breaking by the compensated noncollinear magnetic order. As shown in Figure S13d, a larger ratio $\mid b_{in}/a_{sk}\mid$ indicates a more dominant intrinsic Berry curvature contribution over skew scattering, directly reflecting the enhancement of Berry curvature in Mn_3.5_Pt.
